# Neuroimmunologie von COVID‑19

**DOI:** 10.1007/s00115-021-01077-1

**Published:** 2021-03-02

**Authors:** Thomas Skripuletz, Nora Möhn, Christiana Franke, Harald Prüß

**Affiliations:** 1 Klinik für Neurologie, Medizinische Hochschule Hannover, Hannover, Deutschland; 2 Klinik für Neurologie und Experimentelle Neurologie, Charité – Universitätsmedizin Berlin, Charitéplatz 1, 10117 Berlin, Deutschland; 3 Deutsches Zentrum für Neurodegenerative Erkrankungen (DZNE) Berlin, Berlin, Deutschland

**Keywords:** Enzephalitis, Myelitis, Autoantikörper, Hyperinflammation, Immuntherapie, Encephalitis, Myelitis, Autoantibodies, Hyperinflammation, Immunotherapy

## Abstract

Zahlreiche neuroimmunologische Krankheitsbilder wie Enzephalopathien, Enzephalitiden, Myelitiden oder ADEM (akute disseminierte Enzephalomyelitis) sind nach einer Infektion mit SARS-CoV‑2 („severe acute respiratory syndrome coronavirus 2“) gehäuft aufgetreten, was für einen para- oder postinfektiösen Zusammenhang spricht. Ursächlich ist wahrscheinlich eine virusgetriggerte Überaktivierung des Immunsystems mit Hyperinflammation und Zytokin-Sturm, aber möglicherweise auch die Bildung spezifischer Autoantikörper gegen Gewebe des Zentralnervensystems, die sich vor allem im Liquor schwerkranker COVID-19(„coronavirus disease 2019“)-Patienten finden lassen. Eine direkte Schädigung durch die Invasion von SARS-CoV‑2 ins Gehirn oder Rückenmark scheint keine relevante Rolle zu spielen. Bei Patienten mit Multipler Sklerose, Myasthenie oder anderen neuroimmunologischen Krankheitsbildern wird die Anfälligkeit für eine SARS-CoV-2-Infektion sowie das Risiko eines schweren Verlaufs nicht durch die immunmodulierende Therapie bestimmt, sondern durch bekannte Risikofaktoren wie Alter, Komorbiditäten und den krankheitsbedingten Grad der Behinderung. Immuntherapien sollten bei diesen Patienten daher nicht verschoben oder pausiert werden. Inwieweit neuroimmunologische Mechanismen auch für Langzeitfolgen nach überstandener COVID-19-Erkrankung – wie Fatigue, Gedächtnis‑, Schlaf- oder Angststörungen – verantwortlich sind, werden klinische Verlaufsuntersuchungen u. a. in COVID-19-Registerstudien zeigen.

## Abstract (en)

Many neuroimmunological diseases, such as encephalopathy, encephalitis, myelitis and acute disseminated encephalomyelitis (ADEM) have occurred more frequently after infections with severe acute respiratory syndrome coronavirus 2 (SARS-CoV-2), which indicates a parainfectious or postinfectious association. The most likely underlying mechanisms include virus-triggered overactivation of the immune system with hyperinflammation and cytokine storm but potentially also the development of specific autoantibodies against central nervous system (CNS) tissue. These were predominantly detected in the cerebrospinal fluid of severely ill coronavirus disease 2019 (COVID-19) patients. In contrast, direct damage after invasion of SARS-CoV‑2 into the brain and spinal cord does not seem to play a relevant role. Susceptibility to infection with SARS-CoV‑2 in patients with multiple sclerosis, myasthenia or other neuroimmunological diseases including the risk for severe disease courses, is not determined by the administered immunotherapy but by known risk factors, such as age, comorbidities and the disease-related degree of disability. Therefore, immunotherapy in these patients should not be delayed or discontinued. The contribution of neuroimmunological mechanisms to long-term sequelae after survival of a COVID-19 illness, such as fatigue, impairment of memory, sleep dysfunction or anxiety, will require long-term clinical follow-up, preferentially in COVID-19 register studies.

## Hintergrund

Im Verlauf der Corona-Pandemie wurde immer deutlicher, dass viele Patienten auch neurologische Beschwerden entwickeln. Darunter sind auch Krankheitsbilder, die durch neuroimmunologische Ursachen entstehen, wie Enzephalitiden, Myelitiden, Meningitiden und demyelinisierende Erkrankungen. In ihrer Entstehung spielen vermutlich sowohl hyperinflammatorische als auch durch spezifische Antikörper vermittelte Mechanismen eine Rolle, während eine direkte Schädigung des Zentralnervensystems (ZNS) durch SARS-CoV‑2 („severe acute respiratory syndrome coronavirus 2“) nachrangig zu sein scheint. Etliche Beschwerden können die pulmonale Akutphase der Erkrankung weit überdauern oder erst im Verlauf dazutreten. Dazu gehören (chronische) Erschöpfung, Konzentrationsstörungen, Aufmerksamkeits- und Gedächtnisstörungen, Veränderung der Stimmung sowie Schlafstörungen, an deren Entstehung neuroimmunologische Mechanismen möglicherweise beteiligt sind. Die zum Teil weitreichenden Beeinträchtigungen für betroffene Patienten erfordern eine zügige und gezielte Diagnostik und Therapie sowie besondere Anforderungen an neurologische Rehabilitation und langfristige Versorgung.

Die aktuelle Arbeit gibt eine Übersicht über die neuroimmunologische Beteiligung bei Patienten mit COVID-19 („coronavirus disease 2019“), aber auch über den Einfluss der Viruserkrankung auf eine bereits existierende neurologische Autoimmunkrankheit. Dazu gehört auch die Frage, ob immunmodulierende oder immunsuppressive Therapien im Falle einer COVID-19-Erkrankung weitergeführt werden können. Die systematische Erfassung von Patienten mit neuroimmunologischen Beschwerden in klinischen Registern wird mittelfristig zu einem verbesserten Verständnis der Krankheitsmechanismen, der Behandlungsmöglichkeiten und der klinischen Verläufe führen.

## Neuroimmunologische Krankheitsbilder bei COVID-19

Eine Beteiligung des zentralen Nervensystems bei akuter Infektion mit SARS-CoV‑2 ist charakteristisch für einen schweren COVID-19-Verlauf [8] und wird insbesondere bei intensivpflichtigen Patienten beobachtet.

### Enzephalopathien, Enzephalitis und Meningitis

In einer retrospektiven Untersuchung von 509 Patienten mit COVID-19 wurde bei 31,8 % aller Patienten eine Enzephalopathie beschrieben, bei Patienten mit einem schweren Verlauf sogar in 84,3 % (113/134 Patienten) aller beatmungspflichtigen Patienten [36]. In dieser Kohorte war das Auftreten einer Enzephalopathie mit einem höheren Lebensalter und einer bereits bestehenden neurologischen oder systemischen Vorerkrankung assoziiert. Patienten mit einer Enzephalopathie wurden dreimal länger stationär behandelt und zeigten einen ungünstigeren Krankheitsverlauf mit erhöhter Sterblichkeitsrate [36]. Eine prospektive Untersuchung einer amerikanischen Kohorte von 4491 Patienten konnte in 6,8 % der Fälle eine Enzephalopathie feststellen und gibt wahrscheinlich eine realistischere Einschätzung der tatsächlichen Inzidenz wieder, da hier bei allen eingeschlossenen Patienten eine fachärztliche neurologische Beurteilung durchgeführt worden war.

Nur selten gelingt der Nachweis von SARS-CoV‑2 im Liquor

Die klinische Präsentation einer Enzephalitis bzw. Meningitis kann der einer Enzephalopathie sehr ähnlich sein und sich ebenfalls durch das Auftreten von Kopfschmerzen, meningitischen Reizzeichen und eines Delirs äußern, gekennzeichnet durch eine Bewusstseinsstörung und begleitende neuropsychiatrische Symptome. Das Delir scheint insbesondere bei geriatrischen Patienten auch als primäres und alleiniges Symptom in der frühen Phase von COVID-19 aufzutreten [32]. Wesentliches Unterscheidungskriterium der Enzephalitis von den zahlenmäßig häufiger beschriebenen Enzephalopathien ist jedoch der Nachweis eines entzündlichen Liquorsyndroms mit Pleozytose sowie fokalen Läsionen in der Bildgebung. Ausgesprochen selten gelang dabei der Nachweis von SARS-CoV‑2 im Liquor [42]. Ferner gab es im Rahmen von COVID-19 fulminant verlaufende Fälle einer akuten hämorrhagisch-nekrotisierenden Enzephalopathie mit passender bildgebender Präsentation, diese sind jedoch selten [50].

### Akute disseminierte Enzephalomyelitis

Mehrere Fallberichte dokumentieren das Auftreten einer akuten disseminierten Enzephalomyelitis (ADEM), häufig bei auch pulmonal schwer betroffenen Patienten und vereinzelt zusammen mit einer Myelitis [38, 46, 47]. Sowohl die disseminierten Veränderungen in der Magnetresonanztomographie (MRT) als auch die Liquorbefunde mit starker Eiweißerhöhung ohne relevante Pleozytose entsprechen dabei den charakteristischen diagnostischen Kriterien einer ADEM, in einigen Fällen auch mit hämorrhagischen Läsionen. Die erhöhte Frequenz von ADEM-Patienten bei COVID-19-Patienten spricht für eine para- oder postinfektiöse Ätiologie [47].

### Myelitis

Akute Myelitiden im Rahmen von COVID-19 sind selten und wurden bisher überwiegend als Fallberichte publiziert [1, 2, 44]. Es wurden sowohl jüngere als auch ältere Patienten mit gleicher Geschlechterverteilung beschrieben, die progrediente Lähmungen in Verbindung mit einer Blasenentleerungsstörung entwickelten. Etwa die Hälfte wies keine weiteren Erkrankungen auf. Bei den meisten Patienten zeigte sich in der spinalen MRT eine langstreckige, mehr als 3 Segmente betreffende T2-Hyperintensität sowohl im zervikalen als auch im thorakalen Myelon. Bis auf wenige Ausnahmen fand sich in der kraniellen MRT-Bildgebung ein unauffälliger Befund und die Analyse des Liquors blieb hinsichtlich des Nachweises oligoklonaler Banden negativ. Obwohl die Läsionen an eine Erkrankung wie die Neuromyelitis-optica-Spektrum-Erkrankung (NMOSD) denken lassen, waren nur vereinzelt Aquaporin-4- oder MOG(Myelinooligodendrozytenglykoprotein)-Antikörper nachweisbar. Auch die Analyse des Liquors ergab bis auf wenige Ausnahmen eine normale Zellzahl oder eine leichte Pleozytose mit vorwiegend lymphozytärer Zellverteilung.

### Therapeutische Interventionen

Aufgrund mangelnder Erfahrung mit SARS-CoV‑2 und der Vorsicht vor möglichen Komplikationen als Folge einer Immunsuppression erfolgten die Therapien sehr heterogen [8]. Bei den genannten neuroimmunologischen Krankheitsbildern sind vor allem Steroide sowohl hoch- als auch niedrigdosiert, intravenöse Immunglobuline (IVIG) und therapeutische Apheresen eingesetzt worden. Bei 3 von 5 Patienten mit schwerer Enzephalitis kam es dadurch zu einer ausgeprägten klinischen Besserung [10].

In einer retrospektiven Fallserie von 5 COVID-19-Patienten mit Enzephalopathie hatten IVIG zu einer klinischen Besserung geführt, insbesondere hinsichtlich der Bewusstseinsstörung, sowie zu einer Besserung in der zusatzdiagnostischen Untersuchung mittels Elektroenzephalographie [43]. Etwa die Hälfte der Patienten mit Myelitiden erholte sich nur unvollständig und benötigte eine weitere Rehabilitation. Wenige Betroffene verstarben an kardialen Komplikationen. Besonders schwer war der Verlauf eines 3‑jährigen Mädchens mit einer langstreckigen Myelitis, welches sich trotz einer Kombinationsbehandlung aus Steroiden, IVIG, Plasmapherese und Rituximab von den schweren Lähmungen nicht erholte [31]. Bei ADEM-Patienten sind einzelne deutliche Besserungen unter intravenösen Steroiden dokumentiert, während andere unter Steroiden und IVIG nur eine minimale Verbesserung zeigten.

## Neuroimmunologische Krankheitsmechanismen

Für die Pathophysiologie der COVID-19-assoziierten neuroimmunologischen Erkrankungen wird das Zusammenspiel mehrerer Faktoren vermutet, insbesondere die Schwere der systemischen Erkrankung, (hyper)inflammatorische Prozesse, Koagulopathie und postinfektiöse Autoimmunmechanismen. Im Gegensatz dazu macht der nur ausnahmsweise gelungene Nachweis von SARS-CoV‑2 mittels PCR („polymerase chain reaction“) in Liquorproben bei Patienten mit schweren neurologischen ZNS-Komplikationen [24] eine direkte ZNS-Schädigung durch die Virusinvasion unwahrscheinlich.

### Fehlregulierte Immunantwort: Hyperinflammation als Ursache der neuroimmunologischen Krankheitsbilder?

Die Mortalität von COVID-19 wird hauptsächlich durch die respiratorische Insuffizienz als Folge eines akuten Lungenversagens bestimmt. Patienten mit schweren oder sogar tödlichen Krankheitsverläufen weisen eine spezielle Konstellation an Zytokinen, Chemokinen und anderen Entzündungsfaktoren im Blut auf. Die Analyse einer der ersten COVID-19-Kohorten aus Wuhan ergab, dass u. a. erhöhte Interkeukin(IL)-2-, IL-7-, MIP(„macrophage inflammatory proteins“)-1-α- und Tumornekrosefaktor(TNF)-α-Konzentrationen mit einem schlechteren Outcome assoziiert waren [27]. Auch erhöhte Ferritin- und IL-6-Werte wurden als Prädiktoren für eine erhöhte Sterblichkeit identifiziert [54]. Ähnliche Veränderungen finden sich bei der sekundären hämophagozytischen Lymphohistiozytose (HLH), einem Hyperinflammationssyndrom, das zu einer fulminanten Hyperzytokinämie mit konsekutivem Multiorganversagen führen kann. Sie wird bei Erwachsenen häufig durch Virusinfektionen ausgelöst [39].

Der Serumspiegel von Akute-Phase-Proteinen steigt rasch an

Auf Grundlage dieser Beobachtungen wurde postuliert, dass die Mortalität von COVID-19 durch die virusinduzierte Hyperinflammation bedingt sein könnte. Für einen Zusammenhang zwischen dem sog. Zytokin-Sturm und neurologischen Symptomen spricht der bei Enzephalopathie, Enzephalitis, ADEM und Myelitis häufig gleichzeitig rasch ansteigende Serumspiegel von Akute-Phase-Proteinen (z. B. C‑reaktives Protein [CRP] und Ferritin; [19]). Auffällig ist der oft normale Liquor mit allenfalls leichter Schrankenfunktionsstörung trotz der beobachteten leptomeningealen Kontrastmittelanreicherungen [24]. Das Fehlen einer Liquorpleozytose schließt also die Möglichkeit einer COVID-19-assoziierten entzündlichen ZNS-Erkrankung nicht aus. Aktuelle Einzelzelluntersuchungen im Liquor zeigten funktionell gestörte Monozyten und immunologisch „erschöpfte“ T‑Zellen als möglichen weiteren Faktor einer eingeschränkten antiviralen Immunantwort [25].

Die periphere Hyperinflammation mit einem Anstieg proinflammatorischer Zytokine kann sich auf das ZNS ferner über eine Erhöhung der Permeabilität der Blut-Hirn-Schranke und die Aktivierung von Mikroglia auswirken [29]. Dadurch wird die funktionelle und strukturelle Integrität der Blut-Hirn-Schranke weiter gestört, was zu persistierender Neuroinflammation, neuronaler Exzitotoxizität und zur Schädigung des neurovaskulären Endothels mit verminderter zerebraler Perfusion führen kann. Ein mögliches Korrelat könnten die punktförmigen Diffusionsstörungen sein, die in der kranialen MRT-Bildgebung von COVID-19-Patienten mit ZNS-Symptomen teilweise nachgewiesen werden können [24].

Die Hyperinflammation und die mit ihr einhergehende beeinträchtigte T‑Zell-Antwort wurde als Angriffspunkt für mögliche COVID-19-Therapien diskutiert. Neben dem positiven Effekt einer Immunsuppression durch Glukokortikoide wurden Therapieansätze mit dem IL-6-Rezeptor-Antikörper Tocilizumab und dem IL-1-Rezeptor-Antagonisten Anakinra evaluiert, allerdings ohne Nachweis einer Besserung. Eine weitere Ergründung der Pathomechanismen bei COVID-19 ist erforderlich, um medikamentös immunsupprimierte Patienten hinsichtlich immunmodulatorischer Therapieansätze beraten zu können.

### Virusgetriggerte Autoantikörperbildung

Die Abwesenheit von SARS-CoV‑2 im Liquor lässt an eine indirekt vermittelte Affektion des ZNS denken, die durch Immunzellen oder Autoantikörper vermittelt sein könnte, gestützt auch durch die teils deutliche klinische Besserung unter therapeutischer Apherese [10, 53]. Die Untersuchung von Liquor mittels eines breiten Panels bekannter ZNS-Autoantikörper gegen intrazelluläre und Oberflächenantigene bei COVID-19-Patienten mit neurologischen Syndromen konnte GD1b- und Caspr2-Antikörper (Ak; [23]) nachweisen. Bei eigenen Untersuchungen einer Patientengruppe mit einer schweren COVID-19-Erkrankung auf der Intensivstation und neurologischen Symptomen zeigte sich im Liquor eine hohe Frequenz von Autoantikörpern. Neben einigen etablierten Antikörpern (z. B. Anti-Yo) fand sich in der indirekten Immunfluoreszenz auf unfixierten murinen Hirnschnitten eine starke Immunglobulin(Ig)-G-Bindung gegen astrozytäre Proteine, Gefäßendothel, perinukleäre Antigene oder Neuropil der Basalganglien, des Hippokampus sowie des Bulbus olfactorius [21]. In ähnlicher Weise konnten andere Arbeitsgruppen Autoantikörper gegen verschiedene ZNS-Antigene bei SARS-CoV-2-infizierten Patienten nachweisen, beispielsweise gegen Neurone im Hippokampus und Kortex [61] oder gegen Fasertrakte im Gehirn [19], wobei die Besserung der Patienten unter Immuntherapie für einen pathophysiologischen Zusammenhang spricht.

Die Entstehung von Autoantikörpern nach einer Virusinfektion ist prinzipiell seit längerem bekannt. Ein besonders gut verstandenes Beispiel aus der Neurologie ist die hohe Inzidenz von N‑Methyl-D-Aspartat(NMDA)-Rezeptor-Enzephalitiden nach einer durchgemachten Herpes-simplex(HSV)-1-Enzephalitis [5, 52]. Prospektive Studien belegen ein Auftreten in fast einem Drittel aller Fälle [6]. Postvirale antineuronale Antikörper sind ferner gegen γ‑Aminobuttersäure(GABA)-(A)-, α‑Amino-3-hydroxy-5-methyl-4-isoxazolpropionsäure(AMPA)- und Dopamin-D2-Rezeptoren sowie gegen bisher unbekannte neuronale Antigene beschrieben worden und das Spektrum der zugrunde liegenden Viren reicht von Epstein-Barr(EBV)- über Varizella-Zoster-Virus (VZV), humanes Herpesvirus 6 (HHV-6) und Hepatitisviren bis hin zur Japan-Enzephalitis Typ B [51].

Einige virusneutralisierende SARS-CoV-2-Ak binden auch an anatomische Gehirnstrukturen

**Figure 1:**
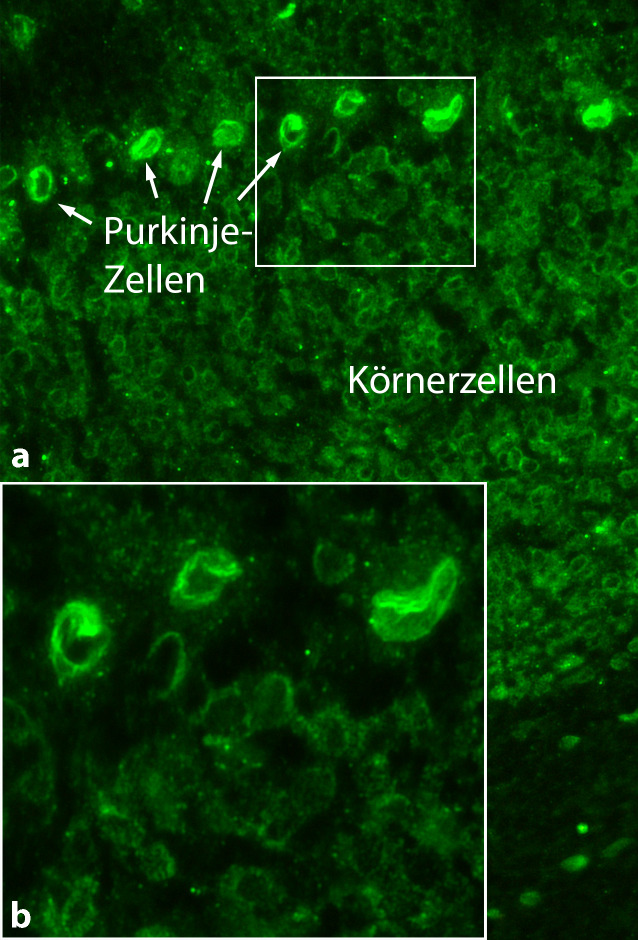

Aufgrund der Vielzahl der Viren und der möglichen postviralen Autoantikörper wird angenommen, dass es zu einer unspezifischen Stimulation von B‑Zellen und Plasmazellen kommt. Plausibel ist die Kostimulation von B‑Zellen durch virale Partikel oder zugrunde gegangenes Hirngewebe über sog. PAMPs („pathogen-associated molecular patterns“; [55]). Ein interessanter neuer Ansatz ist die Möglichkeit eines molekularen Mimikrys, also die Kreuzreaktivität von antiviralen Antikörpern mit Oberflächenstrukturen des eigenen Körpers und Gehirns. So konnte kürzlich in einer Studie gezeigt werden, dass etwa 20 % der hocheffektiv virusneutralisierenden SARS-CoV-2-Antikörper zusätzlich auch an körpereigene Proteine binden [34]. Dabei fiel auf, dass zahlreiche dieser Antikörper gegen wichtige anatomische Strukturen im Gehirn reagierten, beispielsweise gegen Hippokampusneuropil, Astrozyten (Abb. 1) oder zerebrale Gefäße, was gut mit kreuzreaktiven Antikörpern im Sinne des molekularen Mimikrys vereinbar wäre [35]. Ein solcher Krankheitsmechanismus ist bereits für andere neuroimmunologische Erkrankungen beschrieben, allen voran das Guillain-Barré-Syndrom mit der Entstehung kreuzreaktiver postinfektiöser Antikörper gegen Glykolipide auf peripheren Nerven [56], aber auch für nichtneurologische Krankheiten wie nekrotisierende Glomerulonephritiden [30].

Laufende Forschungen werden zeigen müssen, ob diese Antikörper für die neurologischen Beschwerden verantwortlich sind, ob sie sich damit vielleicht sogar als Biomarker neuroimmunologischer Symptome eignen und welche dieser Antikörper für die Einleitung einer Immuntherapie sprechen. Theoretisch erscheint es sogar denkbar, dass kreuzreaktive Autoantikörper nach einer SARS-CoV-2-Impfung entstehen könnten, weshalb die laufenden Impfstudien aus guten Gründen den gleichen strengen Regulierungen unterliegen wie zu Zeiten außerhalb der Pandemie und auf die Entstehung gewebereaktiver Antikörper und entsprechende klinische Symptome achten müssen.

## Erkenntnisse aus vorherigen Coronaviruspandemien: Immunologie und Risikofaktoren

Da insbesondere zu Beginn der aktuellen SARS-CoV-2-Pandemie wenig Daten zu Krankheitsverläufen bzw. zum Infektionsrisiko bei immunsupprimierten Patienten vorlagen und auch aktuell die Datenlage noch unvollständig ist, lohnt sich ein Blick auf die immunologischen Mechanismen im Zusammenhang mit den früheren SARS-1- und MERS(„middle east respiratory syndrome“)-CoV-Pandemien. Als Risikofaktoren für eine Infektion bzw. einen schweren Krankheitsverlauf während der SARS- und MERS-CoV-Ausbrüche ergaben sich ein fortgeschrittenes Alter, männliches Geschlecht und das Vorhandensein von Komorbiditäten (z. B. Adipositas, Diabetes mellitus, Herzerkrankungen, arterielle Hypertonie, Lungenerkrankungen; [60]). Nur in wenigen Studien wurden Patienten mit reduziertem Immunstatus beschrieben [7]. Detaillierte Untersuchungen zu immungeschwächten Patienten und insbesondere zu Patienten unter immunsuppressiver Behandlung fehlen jedoch [18].

Während des ersten SARS-CoV-Ausbruchs 2002 bis 2003, der 916 Todesfälle bei mehr als 8098 infizierten Patienten in 29 Ländern zur Folge hatte, entwickelten die Infizierten eine leichte bis tödliche Lungenerkrankung mit einer Sterblichkeitsrate von mehr als 10 % [49]. Eine eingeschränkte bzw. verzögerte Virusausscheidung als Folge einer suboptimalen T‑ und B‑Zell-Reaktion wurde als Ursache für schwere Krankheitsverläufe postuliert. Patienten mit schlechtem Outcome zeigten eine eingeschränkte humorale Immunität [49]. Gleichzeitig stellte sich eine robuste zelluläre Immunität durch zytotoxische T‑Zellen als protektiv heraus [58]. Studien konnten zeigen, dass epitopspezifische CD8^+^-T-Zellen entscheidend für den Schutz bei einer Reinfektion mit SARS-CoV sind, da die spezifische Antikörperantwort letztlich nur temporär ist [14, 57].

Epitopspezifische CD8^+^-T-Zellen sind wichtig für den Schutz bei einer SARS-CoV-Reinfektion

Das MERS-Coronavirus wurde erstmals 2012 in Saudi-Arabien beschrieben [9]. Die Weltgesundheitsorganisation (WHO) hat seit 2012 in 27 Ländern 2279 Fälle von Infektionen des Menschen mit MERS-Coronavirus bestätigt, wobei die Mortalitätsrate bis Februar 2019 bei 35 % lag. Es hat sich gezeigt, dass MERS-CoV eine Immunsuppression induziert, um der Immunantwort des Wirts zu entgehen, teilweise durch Förderung der T‑Zell-Apoptose. MERS-CoV weist auch Strategien zur Hemmung der angeborenen Immunität und der Interferonproduktion auf [3]. Ähnlich wie SARS-CoV‑1 scheint MERS-CoV eine abgeschwächte angeborene Immunantwort mit verzögerter Induktion proinflammatorischer Zytokine wie Interferon‑γ und IL-12 hervorzurufen [14, 15].

## Umgang mit Immuntherapien während der Pandemie

Eine der Herausforderungen der SARS-CoV-2-Pandemie stellt der Umgang mit chronisch kranken neuroimmunologischen Patienten dar, die einerseits auf eine regelmäßige medizinische Versorgung angewiesen sind, andererseits aber als Risikogruppe zählen, z. B. Patienten mit Multipler Sklerose (MS), Myasthenia gravis oder einer Neuromyelitis-optica-Spektrum-Erkrankung (NMOSD). Wie groß ist dabei das Risiko für diejenigen Patienten, deren Immunsystem medikamentös beeinträchtigt ist, sich mit SARS-CoV‑2 zu infizieren bzw. einen schwereren COVID-19-Verlauf zu erleiden?

### COVID-19-Verläufe bei MS-Patienten: Erkenntnisse aus der Literatur

**Figure 2:**
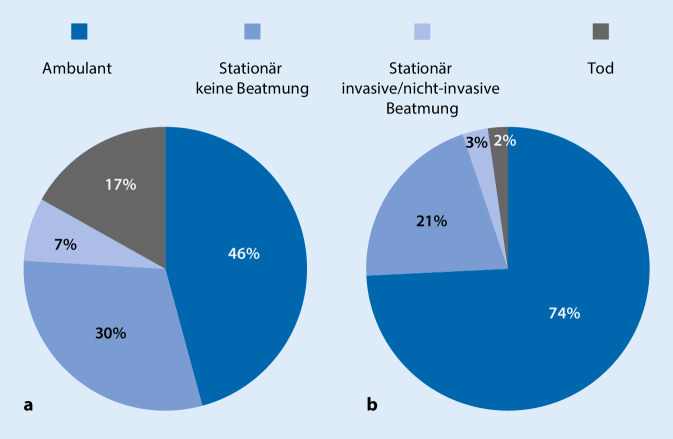

Die Literatur zu MS-Patienten und ihrem potenziellen Risiko im Zusammenhang mit der SARS-CoV-2-Pandemie unterliegt einem Publikationsbias, da mehr Ergebnisse zu Patienten unter hochwirksamen MS-Therapeutika wie der Anti-CD20-Therapie publiziert werden als zu Patienten, die mit Basistherapeutika behandelt werden. Bisher wurden Daten zu insgesamt 873 MS-Patienten publiziert, die an COVID-19 erkrankten bzw. SARS-CoV‑2 positiv waren. Die Sterblichkeitsrate lag bei 4 % [41]. In der Gruppe der Patienten mit Anti-CD20-Therapien betrug die Rate der gemeldeten Todesfälle 3 %, der Anteil schwerer Krankheitsverläufe blieb mit 6 % verhältnismäßig niedrig. Ähnliche oder sogar bessere Outcomeergebnisse wurden für Patienten berichtet, die mit anderen MS-Therapeutika behandelt wurden. Auffällig ist, dass die Gruppe der unbehandelten MS-Patienten im Vergleich ein deutlich schlechteres Outcome zeigt (Abb. 2). Von den 83 bisher publizierten unbehandelten SARS-CoV-2-positiven MS-Patienten starben 17 % an COVID-19 und weitere 7 % benötigten eine Beatmung.

Höheres Alter, (kardiovaskuläre) Komorbiditäten, ein fortgeschrittenes chronisches MS-Erkrankungsstadium und hoher Behinderungsgrad mit Gehunfähigkeit, nicht aber die unterschiedlichen verlaufsmodifizierenden Therapien selbst, wurden in mehreren Studien als Risikofaktoren für eine kritische COVID-19-Erkrankung ggf. mit tödlichem Verlauf identifiziert. Die Daten sprechen dafür, dass unbehandelte und schwer betroffene MS-Patienten besonders gefährdet sind, während die immunmodulierende bzw. immunsuppressive Therapie nicht die entscheidende Gefahr darstellt. Der konkrete Fall einer jungen, ansonsten gesunden MS-Patientin, die unmittelbar nach einer Alemtuzumab-Infusion lediglich einen milden Verlauf von COVID-19 hatte [12], unterstreicht, dass selbst hochwirksame Therapien nicht zwingend zu schweren Krankheitsverläufen führen.

### Immuntherapie bei MS-Patienten fortführen?

Die Befunde zu den früheren Coronaviruspandemien als auch zu SARS-CoV‑2 sprechen dafür, dass kein erhöhtes Infektionsrisiko für Patienten unter Immuntherapien besteht [41] und bisher keine relevant erhöhten Mortalitätsraten bei immunsupprimierten Patienten zu verzeichnen sind [40]. Die Untersuchung von SARS-CoV-2-Antikörpern bei italienischen Patienten mit MS ergab im Vergleich zur Normalbevölkerung ein ähnliches Risiko, sich mit dem Virus zu infizieren [11]. Dies kann aber damit zusammenhängen, dass sich die betroffenen Patienten als Risikogruppe verstehen und grundsätzlich besser schützen. Bezüglich der Krankheitsschwere fällt auf, dass unter den neurologischen Patienten mit immunmodulatorischen Therapien insbesondere diejenigen gefährdet sind, die an (kardiovaskulären) Komorbiditäten leiden oder ein fortgeschrittenes Stadium der neurologischen Grunderkrankung aufweisen. Es gilt daher generell, die chronischen neuroimmunologischen Krankheiten bestmöglich zu behandeln, um Behinderung zu vermeiden [8, 63]. Bei Patienten, die unter ihrer Immuntherapie einen stabilen Erkrankungsverlauf aufweisen, sollte die Behandlung generell nicht pausiert oder modifiziert werden. Speziell für Patienten mit MS gibt die Deutsche Multiple Sklerose Gesellschaft (DMSG) detaillierte Empfehlungen zur Immuntherapie heraus [62].

Da die Hyperaktivität des Immunsystems als Folge der SARS-CoV-2-Infektion möglicherweise mehr Schaden verursacht als das Virus selbst [13, 22], könnten verlaufsmodifizierende MS-Therapien evtl. sogar einen schweren Krankheitsverlauf günstig beeinflussen. So konnte beispielsweise die Anwendung von Inhibitoren des Small-GTPase-RhoA-Signalwegs die Infiltration von Makrophagen verhindern und die Lungenentzündung hemmen [33]. Die MS-Therapeutika Fingolimod und Siponimod sind in der Lage, das Recycling und die Expression von RhoA und RhoA/Aktin-abhängigen Makrophagenrezeptoren zu hemmen, und könnten somit potenziell ein ARDS („acute respiratory distress syndrome“) abschwächen [33]. Teriflunomid verringert die Freisetzung von proinflammatorischen Zytokinen wie IL‑6, IL‑8 und dem von Monozyten stammenden chemotaktischen Protein‑1 (MCP-1) und könnte somit ebenfalls einen eher positiven Effekt im Rahmen einer SARS-CoV-2-Infektion haben.

### Erkenntnisse bei anderen neurologischen Autoimmunerkrankungen

Bisher veröffentlichte Studien zu anderen neurologischen Autoimmunerkrankungen wie der NMOSD oder der Myasthenia gravis zeigen ähnliche Ergebnisse. In größeren NMOSD-Kohorten in unterschiedlichen Ländern lag die SARS-CoV-2-Infektionsrate zwischen 0,07 und 6,7 % [20, 59]. Trotz fortgesetzter (Anti-CD20-)Immuntherapie war der COVID-19-Erkrankungsverlauf in der deutlichen Mehrzahl der Fälle mild [16, 17, 37]. Bei Myastheniepatienten stellt ebenfalls nicht die Immuntherapie selbst eine Gefahr dar, sondern die mögliche Einschränkung der Atemmuskulatur durch die Grunderkrankung. Das Outcome der publizierten Fälle von SARS-CoV-2-positiven Patienten ist variabel [4, 28]. Schwerere Myasthenieverläufe waren erneut vor allem bei Patienten mit weiteren (kardiovaskulären) Vorerkrankungen zu beobachten. Zudem kam es bei einzelnen Patienten zu einer Verschlechterung der Myasthenie unter COVID-19, sodass zusätzliche immunmodulatorische Therapien u. a. mit Steroiden, IVIG oder auch Tocilizumab nötig wurden. Daher steht auch für Myastheniepatienten die Kontrolle der Grunderkrankung im Vordergrund, dazu scheint die Immuntherapie unerlässlich [63].

## Besseres Verständnis der Neuroimmunologie bei COVID-19

### Patientenregister

Seit Beginn der Pandemie im Frühjahr 2020 entstanden Register zur Erfassung neurologischer Symptome bei bislang nichtneurologisch erkrankten Patienten und Patienten mit neurologischer Vorerkrankung und Sars-CoV-2-Infektion. Hierzu zählen im neuroimmunologischen Kontext die Auswertungen des Registers Lean European Observatory on SARS-CoV‑2 Infected Patients (LEOSS) hinsichtlich neuroimmunologisch vorerkrankter Patienten unter Beteiligung des Kompetenznetzes Multiple Sklerose (KKNMS; [48]). Mit ersten Ergebnissen hinsichtlich COVID-19-Verläufen bei neuroimmunologisch Vorerkrankten ist Anfang 2021 zu rechnen. Das Register „Pandemic“ (Pooled Analysis of Neurologic DisordErs Manifesting in Intensive care COVID-19) der Deutschen Gesellschaft für Neurointensiv- und Notfallmedizin (DGNI) hat sich zur Aufgabe gemacht, intensivpflichtige COVID-19-Patienten besser zu charakterisieren. Hier werden interessante Ergebnisse zu neuroimmunologischen Syndromen bei Patienten mit schwer verlaufender SARS-CoV-2-Infektion erwartet [64]. Das Register NAPKON (Nationales Pandemie Kohorten Netz) erfasst fächerübergreifend Patienten mit SARS-CoV-2-Infektionen und erfasst auch neurologische Symptome [45].

### Neurologie und Post-COVID-19

Mittlerweile dokumentieren zahlreiche Studien anhaltende Beschwerden nach COVID-19, die auch nach mildem Verlauf auftreten können. Neurologische Beschwerden umfassen Gedächtnisstörungen, Kopfschmerzen sowie anhaltende Geruchs- und Geschmacksstörungen. Chronische Erschöpfung und Fatigue, Angst, Gelenk- und Muskelschmerzen werden von zahlreichen Patienten berichtet und können neurologischer Genese sein. Nicht primär neurologische Beschwerden wie anhaltende (belastungsabhängige) Luftnot, retropharyngeales Brennen, Husten, Herzstolpern/‑rasen und retrosternales Druckgefühl und Enge bedürfen weiterführender pulmologischer bzw. kardiologischer Abklärung. Es ist derzeit noch nicht absehbar, welche dieser potenziellen Langzeitfolgen auf die oben beschriebenen neuroimmunologischen Veränderungen zurückzuführen sind. Die Analogie zu anderen Viruserkrankungen mit postviralen/postenzephalitischen neurologischen Beschwerden und neuropsychiatrischen Auffälligkeiten lässt einen Zusammenhang möglich erscheinen. Bleibt zu hoffen, dass angesichts der guten medizinischen Versorgung, der zu erwartenden Verfügbarkeit von Impfstoffen und der geringeren Virulenz von SARS-CoV‑2 verglichen mit dem Influenzavirus der „Spanischen Grippe“ von 1918 das Auftreten postviraler Beschwerden wie Parkinsonismus, Dystonien, Fatigue und „Encephalitis lethargica“ deutlich geringer ausfällt als damals mit fast einer Million Betroffene im Jahrzehnt nach dem Ausbruch [26].

Derzeit bilden sich an mehreren deutschen Kliniken Post-COVID-Ambulanzen, die Anlaufstelle für ratsuchende Patienten mit „Neuro-COVID“ sein können. Nach unserer Auffassung sollten Neurologen von Anfang an in diese interdisziplinären Sprechstunden involviert sein oder – wie z. B. an der Charité – eigene spezialisierte neurologische Post-COVID-Sprechstunden anbieten. Mehrjährige strukturierte Nachbetreuungen der Patienten werden notwendig sein, um Langzeitfolgen valide zu erfassen, den Einfluss von Impfungen auf neuropsychiatrische Beschwerden zu verstehen und die Rolle von Immuntherapien auf die Infektionsrate, Symptomschwere und den Verlauf zu klären.

## Fazit für die Praxis

- Neuroimmunologische Krankheitsbilder des Zentralnervensystems (ZNS) bei COVID-19 („coronavirus disease 2019“) umfassen Enzephalitiden, Enzephalopathien, akute disseminierte Enzephalomyelitis und Myelitiden.
- Die überschießende Aktivierung des Immunsystems mit „Hyperinflammation“ und „Zytokin-Sturm“ dominiert die Erkrankungskaskade, während direkter Schaden durch eine Virusinvasion ins ZNS nachrangig zu sein scheint.
- Bei etlichen, vor allem schwer kranken COVID-19-Patienten mit neurologischen Symptomen wurden Autoantikörper in Blut und Liquor nachgewiesen.
- Bei einem Teil der Patienten kann eine Therapie mit Steroiden, intravenösen Immunglobulinen oder therapeutischer Apherese zu einer Besserung führen.
- Das Risiko bei Patienten mit MS und anderen neuroimmunologischen Erkrankungen für einen schweren COVID-19-Verlauf scheint durch Alter, Komorbiditäten und den Grad der Einschränkungen, aber nicht durch die Immuntherapie bestimmt zu werden. In der Regel ist bei diesen Patienten daher keine Änderung der immunmodulierenden Therapie sinnvoll.
